# Comparison of survival in elderly patients treated with uretero-cutaneostomy or ileal conduit after radical cystectomy

**DOI:** 10.1186/s12877-020-01861-9

**Published:** 2021-01-13

**Authors:** Shang Huang, Hanzhong Chen, Teng Li, Xiaoyong Pu, Jiumin Liu, Xuecheng Bi

**Affiliations:** Department of Urology, Guangdong Provincial People’s Hospital, Guangdong Academy of Medical Sciences, No.106, Zhongshan 2nd Road, Yuexiu District, Guangzhou, Guangdong China

**Keywords:** Urinary bladder neoplasms, Urinary diversion, Aged, 80 and over, SEER program

## Abstract

**Background:**

In bladder cancer patients with age ≥ 80 years old, there have been controversies in performing uretero-cutaneostomy or ileal conduit as urinary diversion after radical cystectomy. Limited study evaluated overall survival (OS) and cancer-specific survival (CSS) between the two urinary diversions in elderly patients. This study is to compare OS and CSS between uretero-cutaneostomy and ileal conduit after radical cystectomy in bladder cancer patients with age ≥ 80 years old.

**Patients and methods:**

Data were extracted from the Surveillance, Epidemiology, and End Results (SEER) database. Bladder cancer patients diagnosed between 2004 and 2016 with age ≥ 80 years old who underwent radical cystectomy with either UC or IC were selected. After propensity score matching, Cox regression and Kaplan-Meier analysis were used to analyze the survival. We calculated statistical power for survival.

**Results:**

Of 1394 patients who met the inclusion criteria, 1093 underwent ileal conduit and 301 underwent uretero-cutaneostomy. After propensity score matching, 285 patients were included in each group. Multivariable Cox analysis showed urinary diversion was not a risk factor of OS and CSS (HR 1.044, [95% CI 0.867–1.257] and 1.012 [0.748–1.368], respectively). Both OS and CSS were not significantly different, with median survival of ileal conduit and uretero-cutaneostomy were 19 [16–24] months and 19 [15–26] months respectively. Additionally, We found OS had the following risk factors: tumor stage (distant vs regional vs localized, 5.332 [3.610–7.875] vs 1.730 [1.375–2.176] vs 1), node density (>0.2 vs ≤0.2 vs none, 1.410 [1.047–1.898] vs 0.941 [0.658–1.344] vs 1) and age (1.067 [1.032–1.103] for each year). While CSS had the following risk factors: tumor stage (distant vs regional vs localized, 4.035 [2.046–7.959] vs 2.476 [1.651–3.713] vs 1), node density (>0.2 vs ≤0.2 vs none, 2.501 [1.645–3.804] vs 1.062 [0.590–1.914] vs 1) and tumor size (greater than 3 cm vs less than 3 cm, 1.596 [1.057–2.412] vs 1). Our analysis obtained 0.707 power for overall survival.

**Conclusion:**

Urinary diversion by uretero-cutaneostomy or by ileal conduit was not associated with overall and cancer-specific survival. It is reasonable to consider uretero-cutaneostomy as a regular procedure of urinary diversion in elderly bladder cancer patients after radical cystectomy to avoid associate complications.

## Introduction

Radical cystectomy is one of the most effective treatments to improve survival in bladder cancer patients [1], and consequently becomes first-line procedure of muscle-invasive bladder cancer. Although orthotopic neobladder provides better function than ileal conduit (IC) or uretero-cutaneostomy (UC), more complications occur [2]. Given that continence is inferior in elderly patients who underwent orthotopic neobladder [3] and UC has similar health-related quality of life (HRQoL) due to lower complication rate and to lower expectations [4], UC and IC are more commonly used than continent urinary diversion in elderly patients after removal of urinary bladder [5].

UC is the simplest form of urinary diversion which directly anastomose bilateral ureters to the skin. While IC is more complicated since gastrointestinal segments are detached from digestive tract to form the conduit, and the partitioned digestive tract are reconnected. Due to the smaller diameter of ureters, stricture at skin level is more frequent in UC [6]. Thus, many centers choose IC as standard procedure of urinary diversion in patients who are not suitable for orthotopic neobladder.

However, elderly patients present more comorbidities and shorter life expectancy. Significantly lower survivorship for elderly is observed. Each age increasing leads to 0.2% higher risk of death [7]. Comorbidity in elderly patients is a predictor of cancer-independent mortality [8], leading to shortened overall survival (HR 1.26, *p* = 0.016) [9]. Furthermore, compared with IC, operating time is significantly shorter for UC [10]. Longer operating time is an independent risk factor of Clavien III-V complications (OR 1.005, 95% CI 1.002–1.007 per minute) [11], and Clavien III-V complications is an independent predictor of high cost (OR 12.7, 95% CI 9.63–16.8, [12]). Besides operating time, UC has a shorter stay in intensive care unit (0.9 vs. 2.2 days, *p* < 0.001), and lower Clavien III-V complications (2.1% vs. 14.2%, *p* = 0.01) compared with IC in patients with age ≥ 75 years old [11, 13], and will not significantly impact quality of life of elderly patients [10]. Consequently, UC is more likely to be performed in high-risk elderly patients [3].

Most studies comparing UC and IC focused on complications and early mortality. Given that UC incurs less perioperative and postoperative complications than IC but more late complications such as ureteral obstruction at skin level, balance of benefits, risks and life expectancy should be emphasized in elderly patients. Based on evidences mentioned above, we hypothesize UC would have similar overall survival and cancer- specific survival compared with IC. This research is the first time to evaluate long-term survival of UC and IC in patients with age ≥ 80 years old and will provide further information on urinary diversion selection.

## Methods

### Study population and grouping

Data were extracted from the Surveillance, Epidemiology, and End Results (SEER) database [14]. Data extraction and analysis were completed from November, 2019 to Februrary, 2020. The inclusion criteria are listed below:

1. Age at diagnosis≥80 years old.
2. Diagnosed between 2004 and 2016.
3. Had microscopically diagnosed bladder cancer.
4. Underwent radical cystectomy with either UC or IC.

We included all histologic types of bladder cancer. Sociodemographic and clinical characteristics were selected as baseline data. In detail, we selected age, sex, marital status, year of diagnosis, race/ethnicity, insurance, tumor grade, tumor stage, tumor size, and node density. Marital status was categorized as ‘Married’, ‘Single’, and ‘Unknown’. We combined all living-alone status into ‘Single’. Tumor grade was categorized as ‘Urothelial Carcinoma, Low Grade’, ‘Urothelial Carcinoma, High Grade’, ‘Urothelial Carcinoma, NOS’ (NOS indicated not otherwise specified), and ‘non-Urothelial Carcinoma’. We used ‘SEER Summary Stage 2000’ as a comprehensive interpretation to TNM stage system. In detail, tumor stage was categorized as ‘Localized’ which means Tis ~ T2bN0M0, ‘Regional’ which means T3a ~ T4N0M0 or TanyN1 ~ N2M0, ‘Distant’ which means TanyN3M0 or TanyNanyM1, and ‘Unknown’. Tumor size was categorized as ‘Less than 3cm’, ‘Greater than 3cm’, and ‘Unknown’. Node density was calculated as nodes positive divided by nodes examined after surgery. If no node was positive, node density was categorized as ‘None’. If any nodes were positive, node density was categorized as ‘≤0.2’ and ‘>0.2’. If unknown nodes were positive, node density was categorized as ‘Unknown’.

### Statistical analysis

Patients were divided into IC or UC group according to urinary diversion they had received. We used propensity score matching with 1:1 ratio by baseline characteristics stated above as pseudo randomization. All statistical analysis used data after matching. We performed power analysis for survival to calculate the statistical power of this study according to Freedman et al. [15]. Little information was provided for postulated hazard ratio from previous studies, so we set postulated hazard ratio in a post hoc manner.

Risk factors were determined using Cox proportional hazardous regression model. The primary outcome is overall survival (OS). The secondary outcome is cancer-specific survival (CSS). OS and CSS were evaluated by Kaplan-Meier method with Log-rank test. The Analysis of Variance or Kruskal-Wallis test was used to analyze continuous variables. Chi-square test or Fisher’s exact test was used to analyze categorical variables. All statistical tests were 2-sided. *p*-value< 0.05 was considered statistically significant. R software version 3.6.3 with packages survival, survminer, MatchIt, and powerSurvEpi was used to perform calculation and graphing.

## Results

### Clinical and sociodemographic characteristics

Of 1394 patients who met the inclusion criteria, median age was 82.0 (interquartile range 81.0–85.0), with 1206 (86.5%) were men and 188 (13.5%) were women. Before matching, 1093 patients were included in IC group and 301 patients were included in UC group. IC group had fewer male patients (83.8% vs 96.3%, *p* < 0.001), later year of diagnosis (between 2004 and 2010, 60.2% vs 76.7%, *p* < 0.001), more insured patients (76.0% vs 63.8%, *p* < 0.001) and different node density (*p* = 0.021) than UC group. While age, marital status, race/ethnicity, tumor grade, tumor stage and tumor size had no significant differences between two groups. After matching, 285 patients were in each group. All sociodemographic and clinical characteristics had no significant differences. Patient demographics before and after propensity score matching are summarized in Table 1.

**Table 1:** Patient demographics before and after propensity score matching

	Ileal Conduit	Uretero-cutaneostomy	p
Before Matching			
n	1093	301	
Age (median [IQR])	82.00 [81.00, 85.00]	83.00 [81.00, 85.00]	0.596
Sex (n (%))			< 0.001
Female	177 (16.2)	11 (3.7)	
Male	916 (83.8)	290 (96.3)	
Marital Status (n (%))			0.132
Married	695 (63.6)	187 (62.1)	
Single	361 (33.0)	96 (31.9)	
Unknown	37 (3.4)	18 (6.0)	
Year of Diagnosis (n(%))			< 0.001
2004 ~ 2010	658 (60.2)	231 (76.7)	
2011 ~ 2016	435 (39.8)	70 (23.3)	
Race/Ethnicity (n(%))			0.940
White	995 (91.0)	273 (90.7)	
Black	48 (4.4)	13 (4.3)	
Asian	50 (4.6)	15 (5.0)	
Insurance (n(%))			< 0.001
Insured	831 (76.0)	192 (63.8)	
Uninsured	6 (0.5)	1 (0.3)	
Unknown	256 (23.4)	108 (35.9)	
Tumor Grade (n (%))			0.261
Urothelial Carcinoma, Low Grade	33 (3.0)	16 (5.3)	
Urothelial Carcinoma, High Grade	946 (86.6)	252 (83.7)	
Urothelial Carcinoma, NOS	15 (1.4)	5 (1.7)	
non-Urothelial Carcinoma	99 (9.1)	28 (9.3)	
Tumor Stage (n (%))			0.159
Localized	398 (36.4)	123 (40.9)	
Regional	620 (56.7)	151 (50.2)	
Distant	72 (6.6)	26 (8.6)	
Unknown	3 (0.3)	1 (0.3)	
Tumor Size (n (%))			0.169
Less than 3 cm	262 (24.0)	68 (22.6)	
Greater than 3 cm	508 (46.5)	127 (42.2)	
Unknown	323 (29.6)	106 (35.2)	
Node Density (n(%))			0.021
None	611 (55.9)	160 (53.2)	
≤ 0.2	133 (12.2)	22 (7.3)	
>0.2	117 (10.7)	38 (12.6)	
Unknown	232 (21.2)	81 (26.9)	
After Matching			
n	285	285	
Age (median [IQR])	82.00 [81.00, 84.00]	83.00 [81.00, 85.00]	0.689
Sex (n (%))			0.835
Female	13 (4.6)	11 (3.9)	
Male	272 (95.4)	274 (96.1)	
Marital Status (n (%))			0.818
Married	183 (64.2)	180 (63.2)	
Single	93 (32.6)	93 (32.6)	
Unknown	9 (3.2)	12 (4.2)	
Year of Diagnosis (n(%))			0.128
2004 ~ 2010	231 (81.1)	215 (75.4)	
2011 ~ 2016	54 (18.9)	70 (24.6)	
Race/Ethnicity (n(%))			0.655
White	258 (90.5)	262 (91.9)	
Black	11 (3.9)	12 (4.2)	
Asian	16 (5.6)	11 (3.9)	
Insurance (n(%))			0.895
Insured	186 (65.3)	190 (66.7)	
Uninsured	1 (0.4)	1 (0.4)	
Unknown	98 (34.4)	94 (33.0)	
Tumor Grade (n (%))			0.967
Urothelial Carcinoma, Low Grade	15 (5.3)	13 (4.6)	
Urothelial Carcinoma, High Grade	241 (84.6)	240 (84.2)	
Urothelial Carcinoma, NOS	5 (1.8)	5 (1.8)	
non-Urothelial Carcinoma	24 (8.4)	27 (9.5)	
Tumor Stage (n (%))			0.851
Localized	106 (37.2)	116 (40.7)	
Regional	154 (54.0)	146 (51.2)	
Distant	24 (8.4)	22 (7.7)	
Unknown	1 (0.4)	1 (0.4)	
Tumor Size (n (%))			0.581
Less than 3 cm	74 (26.0)	67 (23.5)	
Greater than 3 cm	127 (44.6)	123 (43.2)	
Unknown	84 (29.5)	95 (33.3)	
Node Density (n(%))			0.408
None	166 (58.2)	155 (54.4)	
≤ 0.2	24 (8.4)	22 (7.7)	
>0.2	38 (13.3)	34 (11.9)	
Unknown	57 (20.0)	74 (26.0)	

*NOS* Not Otherwise Specified, *IQR* Interquartile Range. Node density is nodes positive divided by nodes examined after surgery

### Risk factor of OS and CSS

Multivariable Cox proportional hazardous regression model showed that UC was associated with neither OS nor CSS (HR 1.044, 95% CI 0.867–1.257 and HR 1.012, 95% CI 0.748–1.368, respectively). But tumor stage and node density were risk factors for both OS and CSS. Compared to localized tumor, regional and distant involvement had decreased OS (HR 1.730, 95% CI 1.375–2.176 and HR 5.332, 95% CI 3.610–7.875), as well as CSS (HR 2.476, 95% CI 1.651–3.713 and HR 4.035, 95% CI 2.046–7.957). Compared to zero node density (‘None’), > 0.2 node density had decreased OS (HR 1.41, 95% CI 1.047–1.898), as well as CSS (HR 2.501, 95% CI 1.645–3.804). Age increasing had negative correlation with OS (HR 1.067, 95% CI 1.032–1.103 for each year). Tumor size greater than 3 cm had negative correlation with CSS (HR 1.596, 95% CI 1.057–2.412). Test of the proportional hazard assumption based on Schoenfeld residuals showed *p* > 0.05 for all covariates. Thus, all sociodemographic and clinical characteristics were taken into consideration during Cox analysis. The multivariable Cox analysis results are summarized in Table 2.

**Table 2:** Multivariable Cox proportional hazardous regression model for overall survival and cancer-specific survival

Characteristics	Hazard Ratio [95% CI]	p
Overall Survival		
Urinary Diversion		
Ileal Conduit	1 [Reference]	
Uretero-cutaneostomy	1.044 [0.867–1.257]	0.651
Age	1.067 [1.032–1.103]	< 0.001
Sex		
Female	1 [Reference]	
Male	0.857 [0.521–1.411]	0.545
Marital Status		
Married	1 [Reference]	
Single	1.018 [0.828–1.251]	0.867
Unknown	1.039 [0.593–1.822]	0.894
Year of Diagnosis		
2004 ~ 2010	1 [Reference]	
2011 ~ 2016	0.923 [0.700–1.217]	0.569
Race/Ethnicity		
White	1 [Reference]	
Black	0.894 [0.555–1.440]	0.644
Asian	0.911 [0.574–1.445]	0.692
Insurance		
Insured	1 [Reference]	
Uninsured	1.136 [0.156–8.259]	0.900
Unknown	1.005 [0.814–1.241]	0.963
Tumor Grade		
Urothelial Carcinoma, Low Grade	1 [Reference]	
Urothelial Carcinoma, High Grade	1.228 [0.777–1.941]	0.379
Urothelial Carcinoma, NOS	1.024 [0.461–2.276]	0.953
non-Urothelial Carcinoma	1.533 [0.891–2.638]	0.123
Tumor Stage		
Localized	1 [Reference]	
Regional	1.730 [1.375–2.176]	< 0.001
Distant	5.332 [3.610–7.875]	< 0.001
Unknown	3.017 [0.679–13.411]	0.147
Tumor Size		
Less than 3 cm	1 [Reference]	
Greater than 3 cm	1.185 [0.930–1.510]	0.170
Unknown	1.253 [0.969–1.619]	0.085
Node Density		
None	1 [Reference]	
≤ 0.2	0.941 [0.658–1.344]	0.737
>0.2	1.410 [1.047–1.898]	0.024
Unknown	1.487 [1.167–1.895]	0.001
Cancer-Specific Survival		
Urinary Diversion		
Ileal Conduit	1 [Reference]	
Uretero-cutaneostomy	1.012 [0.748–1.368]	0.940
Age	0.983 [0.928–1.041]	0.561
Sex		
Female	1 [Reference]	
Male	0.795 [0.374–1.690]	0.551
Marital Status		
Married	1 [Reference]	
Single	1.297 [0.934–1.801]	0.120
Unknown	1.308 [0.558–3.065]	0.537
Year of Diagnosis		
2004 ~ 2010	1 [Reference]	
2011 ~ 2016	1.242 [0.828–1.863]	0.294
Race/Ethnicity		
White	1 [Reference]	
Black	0.768 [0.336–1.756]	0.532
Asian	0.795 [0.367–1.724]	0.561
Insurance		
Insured	1 [Reference]	
Uninsured	2.707 [0.354–20.679]	0.337
Unknown	1.118 [0.789–1.583]	0.532
Tumor Grade		
Urothelial Carcinoma, Low Grade	1 [Reference]	
Urothelial Carcinoma, High Grade	0.934 [0.482–1.807]	0.838
Urothelial Carcinoma, NOS	0.688 [0.185–2.556]	0.577
non-Urothelial Carcinoma	1.241 [0.547–2.813]	0.606
Tumor Stage		
Localized	1 [Reference]	
Regional	2.476 [1.651–3.713]	< 0.001
Distant	4.035 [2.046–7.959]	< 0.001
Unknown	3.093 [0.354–27.006]	0.307
Tumor Size		
Less than 3 cm	1 [Reference]	
Greater than 3 cm	1.596 [1.057–2.412]	0.026
Unknown	1.378 [0.872–2.176]	0.17
Node Density		
None	1 [Reference]	
≤ 0.2	1.062 [0.590–1.914]	0.840
>0.2	2.501 [1.645–3.804]	< 0.001
Unknown	1.995 [1.340–2.969]	0.001

*NOS* Not Otherwise Specified, *CI* Confidence Interval. Node density is nodes positive divided by nodes examined after surgery

### Survival comparison between IC and UC

**Fig. 1:**
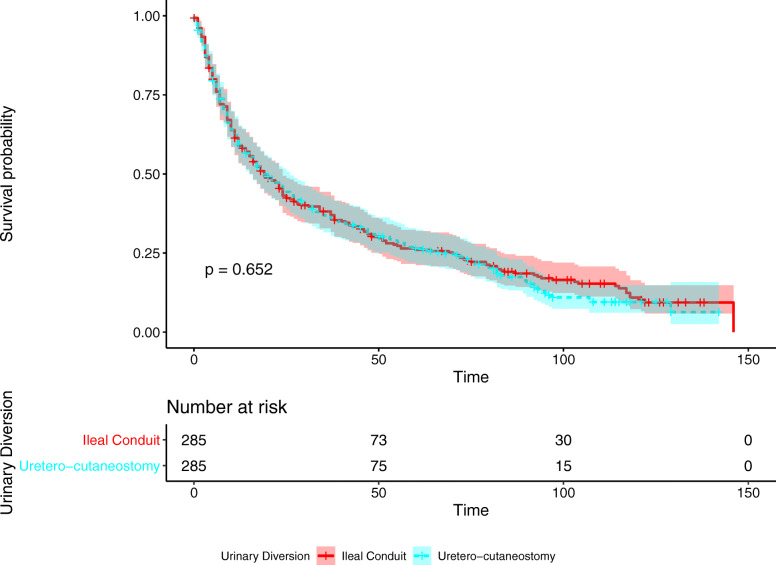
Kaplan-Meier curve of overall survival according to urinary diversion after propensity score matching. No significant difference is found between ileal conduit and uretero-cutaneostomy with median survival 19 months (95% CI 16–24) and 19 months (95% CI 15–26) respectively (*p* = 0.652). Survival is measured in months

**Fig. 2:**
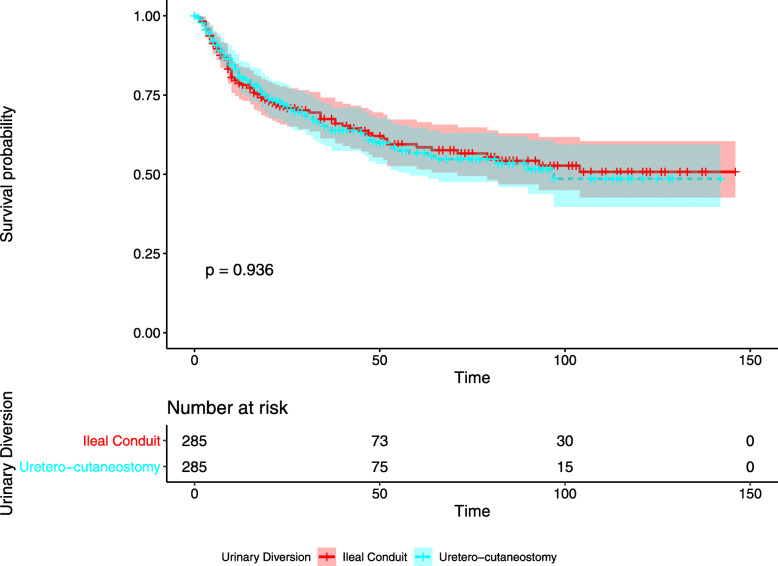
Kaplan-Meier curve of cancer-specific survival according to urinary diversion after propensity score matching. No significant difference is found between ileal conduit and uretero-cutaneostomy (*p* = 0.936). Survival is measured in months

Kaplan-Meier curve of OS showed median survival of IC and UC were 19 months (95% CI 16–24) and 19 months (95% CI 15–26). No significant differences are observed (*p* = 0.652). Furthermore, no significant difference was found in Kaplan-Meier curve of CSS (*p* = 0.936). The two curves are shown in Figs. 1 and 2.

### Power analysis

**Fig. 3:**
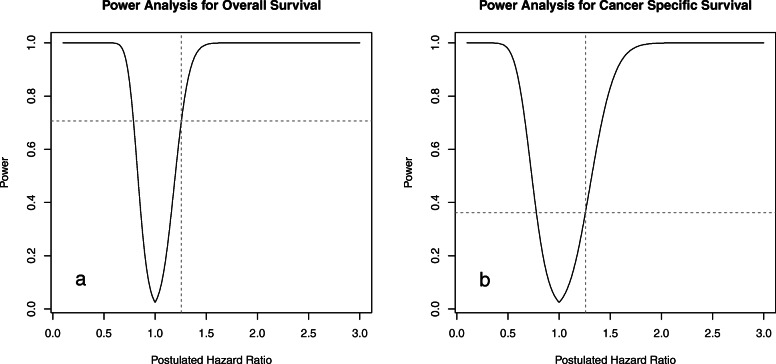
Power-effect curve of overall survival (**a**) and cancer-specific survival (**b**) with *N* = 570 and alpha = 0.05. Vertical dotted line indicates postulated hazard ratio of 1.257; Horizontal dotted line indicates the power; *N* = 570 because 285 patients are in each urinary diversion group

Given that we obtained upper CI of 1.257 in multivariable Cox analysis between IC and UC, we set postulated HR to 1.257 for power analysis. Power analysis showed that we had achieved 0.707 power for OS and 0.362 power for CSS. To reduce ambiguity of power analysis we drew a power-effect curve to clearly show relation between power and postulated HR. The power-effect curve is shown in Fig. 3.

## Discussion

Natural history of bladder cancer is dreadful, with 38% patients develop metastasis after 6 months if untreated, and only 5% patients would survive after 5 years [16, 17]. Radical cystectomy is the standard treatment for muscle invasive bladder cancer. However, it is a challenge to determine the best urinary diversion for different patient populations after radical cystectomy.

Current studies comparing UC and IC mainly focus on perioperative complications and early mortality, and the results vary [18]. In elderly patients especially those with cardiac or pulmonary comorbidities, IC or UC are recommended to reduce complications. UC has shorter operating time, shorter stay in intensive care unit, shorter fasting period, and lower Clavien III-V complications compared with IC in elderly patients [6, 10, 11, 13,19]. While some studies failed to conclude such advantages [20, 21].

For now, few studies have compared UC with IC for OS and CSS, let alone long-term survival. Big data in SEER database gives us a chance to monitor UC and IC. In this study we offered a retrospective analysis based on SEER database. In our study, we used propensity score matching by 10 socioeconomic and clinical characteristics to minimize potential bias. During the matching process, we found newly diagnosed patients who were diagnosed between 2011 ~ 2016 were more likely to undergo IC. This is reasonable because wide spread of laparoscopic and robotic surgery could ease concerns about perioperative complications. However, this shift of surgical technique has not brought us survival benefit yet. According to our analysis. The 95% CI was 0.828–1.863.

After propensity score matching, our results showed IC and UC have similar OS and CSS. In study by Garde et al. [22], UC is not a risk factor compared with IC for OS (HR 1.86, 95% CI 0.69–5.03), but lymph node involvement is (HR 8.56, 95% CI 1.57–46.73). This is consistent with our findings that UC is not a risk factor but tumor stage is. Moreover, we found node density >0.2 could significantly impact OS and CSS, indicating node density could potentially become a predictor of survival in clinical setting. To summarize, it is patients’ somatic function and clinical characteristics but not urinary diversion which determine OS and CSS. Thus, UC could be a regular urinary diversion technique for patients with age ≥ 80 years old.

The main drawback of UC is stricture at skin level. If left ureter has to be connected to right ureter first other than pulled out of right abdominal skin directly, the ureter-ureter anastomosis could also be an obstructive site. Clean intermittent dilation with a disinfectant stick by patient himself could be a solution to skin-level obstruction. Ureter-ureter anastomosis stricture may require long-term ureter stent. Few complications are observed with replacement every 6 months [23]. However, our experience shows that under most circumstances the left ureter is long enough to be fixed at right abdominal skin directly, even in an extraperitoneal way.

Few studies compared survival of IC and UC, therefore little information could be provided on postulated HR. We set postulated HR to 1.257 in power calculation in a post hoc manner because in multivariable Cox analysis the upper CI of UC compared with IC was 1.257. Hence, we gained 0.707 power for OS. We have further calculated that to achieve 0.8 power with 1.1 postulated HR and 0.05 alpha, approximately 2700 patients should be included in each group. The actual number depends on proportion of censored data. To reduce ambiguity of power analysis we drew a power-effect curve to clearly show relation between power and postulated HR. This will help further interpretation of this study.

There are limitations in our study.

Firstly, this study is retrospective with inevitable selection bias. Although we attempt to balance two groups by propensity score matching, some important sociodemographic and clinical characteristics are missing due to lack of data in SEER database. Former studies suggested comorbidity has negative impact on survival [9, 24]. Charlson Comorbidity Index (CCI) may be a better survival predictor than age alone [25, 26]. Such sociodemographic and clinical characteristics should be taken into consideration in future studies.

Secondly, we did not take functional outcomes into analysis. The reason is similar that such outcomes are not recorded in SEER database. Early research showed similar glomerular infiltration rate between IC and continent pouch [27], while UC results in slight loss of renal function at approximately 8 ml/min/1.73m^2^ [19]. Although renal function deterioration is mainly observed in preoperative renal disease patients [28] and may not be a lethal cause in clinical setting, functional outcomes should be evaluated in large cohort in future studies.

## Conclusion

Uretero-cutaneostomy provides similar overall survival and cancer-specific survival compared with ileal conduit. Considering a lower perioperative and postoperative complication rate than ileal conduit, uretero-cutaneostomy could become a regular urinary diversion technique for patients with age ≥ 80 years old.

## Data Availability

The dataset supporting the conclusions of this article is available in the Surveillance, Epidemiology, and End Results (SEER) database. The URL of the database is https://seer.cancer.gov/data.
